# Cellular Crosstalk between Endothelial and Smooth Muscle Cells in Vascular Wall Remodeling

**DOI:** 10.3390/ijms22147284

**Published:** 2021-07-06

**Authors:** Nerea Méndez-Barbero, Carmen Gutiérrez-Muñoz, Luis Miguel Blanco Colio

**Affiliations:** 1 Vascular Research Laboratory, IIS-Fundación Jiménez Díaz University Hospital, Av. Reyes Católicos 2, 28040 Madrid, Spain; carmen.gutierrezm@estudiante.uam.es (C.G.-M.); lblanco@fjd.es (L.M.B.C.); 2 Centro de Investigación Biomédica en Red de Enfermedades Cardiovasculares (CIBERCV), 28029 Madrid, Spain

**Keywords:** vascular wall remodeling, endothelial cells, smooth muscle cells, crosstalk, cocultures

## Abstract

Pathological vascular wall remodeling refers to the structural and functional changes of the vessel wall that occur in response to injury that eventually leads to cardiovascular disease (CVD). Vessel wall are composed of two major primary cells types, endothelial cells (EC) and vascular smooth muscle cells (VSMCs). The physiological communications between these two cell types (EC–VSMCs) are crucial in the development of the vasculature and in the homeostasis of mature vessels. Moreover, aberrant EC–VSMCs communication has been associated to the promotor of various disease states including vascular wall remodeling. Paracrine regulations by bioactive molecules, communication via direct contact (junctions) or information transfer via extracellular vesicles or extracellular matrix are main crosstalk mechanisms. Identification of the nature of this EC–VSMCs crosstalk may offer strategies to develop new insights for prevention and treatment of disease that curse with vascular remodeling. Here, we will review the molecular mechanisms underlying the interplay between EC and VSMCs. Additionally, we highlight the potential applicable methodologies of the co-culture systems to identify cellular and molecular mechanisms involved in pathological vascular wall remodeling, opening questions about the future research directions.

## 1. Introduction

Cardiovascular diseases (CVD) are a general term to define a group of heart and blood vessel disorders that include coronary heart disease, cerebrovascular disease, peripheral arterial disease, and aortic disease. CVD are the main cause of death in developed countries. The rates of CVD incident and case-fatality have fallen considerably over the last two decades in those countries due to the investigation of new therapies and diagnosis. However, nowadays it still accounts for 17.3 million deaths per year, and it is expected to grow to more than 23.6 million by 2030 [1]. In this sense, CVD claims more lives than all forms of cancer combined. Moreover, due to our lifestyle in which we are exposed to many cardiovascular risk factors (such as smoking, hypertension, cholesterol diet, sedentary life style, stress, or pollution), a high percentage of the population have developed an asymptomatic vascular damage, affecting the vascular homeostasis. This high mortality rate indicates the need to identify the molecular mechanisms that occur during the pathogenesis of CVD, in order to develop new strategies for the early diagnosis and treatment to avoid the fatal ending.

The vasculature is one of the first organs to develop during embryogenesis and it is fundamental for the correct function of all other organs. Arteries are essential to maintain vascular tone as they regulate changes in pressure and blood flow due to their contractile nature and the mechanical properties conferred by their wall (elasticity, tensile rigidity, and comprehensibility) [2]. The arterial wall is organized in a structure of three concentric and independent layers of cells interconnected (intima, media, and adventitia), that acts as a functional unit, guaranteeing the integrity and functionality of the vessels [3]. On the innermost side of the vessel wall, the semipermeable layer of endothelium selectively limits the movement of macromolecules [4], and essentially involves in vascular tone, fluid homeostasis, and host defense [5]. Endothelial cells (EC) are exposed to changes in the lumen of the vessels, as mechanical injury, shear stress or chemicals agents. In these circumstances, EC release various cytokines, chemokines, and growth factors that result in endothelial dysfunction phenotype and trigger the progression of cardiovascular diseases such as hypertension, atherosclerosis, aging, stroke, heart disease, diabetes, obesity, venous thrombosis, and intimal hyperplasia [6]. In the medial layer, vascular smooth muscle cells (VSMCs) are the main component, exhibit remarkable phenotypic plasticity and can dedifferentiate from a contractile state to a synthetic state. These phenotypic modifications regulate proliferative, migratory and inflammatory capacities of the VSMCs, which play a major role in arterial remodeling [7]. The communication between EC from the intima layer and VSMCs for the media layer, is a critical step in the initiation and progression of pathological vascular remodeling. Perturbations in the EC–VSMCs communications can lead, for example, to the typical features of atherosclerosis development such as; endothelial dysfunction, inflammatory cell infiltration or phenotypic switching of VSMCs [8].

The vessel wall is continuously exposed to local mechanical, hemodynamic and neurohumoral stimuli such as uncontrolled changes in blood pressure, inflammatory response processes, mechanical damage to the vessels, accumulation of lipids, etc., that elicit adaptive and functional responses. However, when these stimuli are sustained in time, they comprise molecular, cellular, and interstitial changes which, at tissue level, are reflected by modifications in the luminal diameter, the thickness of the wall and the structure of the transverse areas of the media and/or adventitia [9,10]. Vascular wall remodeling refers to the adaptation of the vessel wall to biochemical and biomechanical stimuli [11]. This remodeling is an active process that involves changes in the proliferative and migratory status of VSMCs, endothelial dysfunction, inflammatory processes, as well as synthesis or degradation of the components of the extracellular matrix (ECM) [12]. All these processes are regulated by the dynamic interaction of growth factors, vasoactive substances, and hemodynamic stimuli between cells, which trigger changes in the structure and functionality of the vascular wall [13,14]. These pathological changes in the structure and composition of blood vessels could contribute to the pathophysiology of vascular diseases and circulatory disorders, and cause clinical manifestations [15]. Identifying the pathways underlying EC–VSMCs interaction that mediate vascular homeostasis in the course of vascular remodeling may offer strategic insight for CVD prevention. Vascular wall remodeling is classified in several categories; outward (increase) and inward (decrease) remodeling when the diameter of the lumen varies, with respective hypertrophy (thickening), hypotrophy (thinning) or eutrophic (no change) of the vessel wall [16]. Hypotrophic remodeling results in a thinner vessel wall, with a decrease in the wall-to-lumen ratio, which can be both inward and outward. Hypertrophic remodeling results in the thickening of the vessel wall, with an increase wall-to-lumen ratio, that can also be inward and outward. In the eutrophic situation, wall-to-lumen ratios do not change, but the size of the vessel can differ. Depends on the pathology, they could undergo a different arterial remodeling. In the case of the atherosclerosis for example, it is termed as inward or outward hypertrophic remodeling and is characterized by an increase in vessel diameter with the thickening of both the media and intima [17]. Additionally, the aneurysm formation is characterized by an increase in vessel diameter, with a thinning of the vessel wall, and termed outward hypotrophic remodeling [16]. Inward remodeling is less frequently observed and is seen in more muscular peripheral arteries, probably reflecting the sustained vasoconstriction of vessels [18].

In this review, we summarized the different pathways of cell–cell communication as well as the different in vitro models to analyze EC–VSMCs interaction.

## 2. EC–VSMC Communication

Cell–cell communication between EC and VSMCs plays a critical role not only in vascular homeostasis but also in disease. Under physiological conditions, VSMCs have a contractile phenotype, which facilitates the contraction and dilation of the vasculature that, in smaller resistance arteries, is essential for the regulation of blood flow. The response of blood vessels to physiological and pathological stimuli partly depends on the crosstalk between EC lining the luminal side and VSMCs in the inner part of the vascular wall.

The EC–VSMCs dialogue for the maintenance of vascular homeostasis is established in several ways; by direct cell contact and by indirect interaction via ECM or through soluble secreted molecules and extracellular vesicles (EVs) [19]. In this regard, to maintain the functional contractile state of VSMCs a continuous release of vasoactive compounds from EC (such as prostanoids, arachidonic, acid metabolites, and nitric oxide) is required [20]. However, when a mechanical/chemical injury or loss of the endothelium take place, there is a disturbed crosstalk between EC and VSMCs, that would trigger VSMCs phenotypic and functional changes (from contractile to proliferative), inflammation, and ECM deposition, all of them featuring the pathological vascular wall remodeling [8].

Therefore, this corroborates the reason why the in vitro analysis of the pathophysiology of blood vessels requires coculture systems of EC and VSMCs. For a long time, many research groups have tried to elucidate the functions of ECs and VSMCs as independent entities due to either easy results analysis or lack of established protocols. For that reason, it remains unclear how defects in the EC–VSMC physical or paracrine interaction, or with their microenvironment (extracellular matrix) could lead to CVD and it if will help to identify key players for diagnosis and/or treatment of vascular remodeling diseases.

### 2.1. Paracrine Communications

**- Soluble factors**

Diffusion of soluble factors and balance between their concentrations are an essential process in the EC–VSMCs communications of mature blood vessels. As VSMCs are not directly exposed to the mechanical or chemical changes in the blood flow, the EC-induced signal transduction is the way of communication for VSMCs to react. In a healthy vessel wall, the endothelium is able to regulate and control the growth, and the phenotype of VSMCs releases various vasoactive factors [21]. The majority of the described studies have been focused in the paracrine regulation of vascular tone [22,23,24,25]. However, the role of paracrine EC–VSMCs communication during pathological situations in the mature vasculature needs to be more deeply addressed. Several coculture systems have been developed to study EC–VSMCs interactions, and have demonstrated that the simple interaction of both cells regulates paracrine expression of some molecules. In this respect, coculture of ECs and VSMCs on opposite sides of a transwell membrane, triggers changes in VSMCs phenotype [26] and the up-regulation of different molecules such as, VEGF, PDGF-AA, PDGF-BB, and TGF-β and down-regulation of bFGF [27,28]. Interestingly, cultured ECs with VSMCs also changed ECs morphology, increased EC gene expression of tissue factor [29], VEGF [27], adhesion molecules [30], growth-related oncogene-α and monocyte chemotactic protein-1 [31]. Moreover, paracrine factors released from EC could also be regulators of VSMCs metabolic processes that has been described important in the context of immunometabolism response in the vascular remodeling disease such as atherosclerosis [32]. Some soluble factors from EC have the capacities to change the low density lipoprotein metabolism of VSMCs [33] or reduce cholesteryl ester hydrolysis as compared to solo-cultured VSMCs [34].

In physiological situations, paracrine EC–VSMCs communication is one of the main ways to control vessel contraction. In this sense, endothelial-derived factors such as nitric oxide (NO), prostacyclin, and hyperpolarizing agents diffuse out from endothelial cell to the underlying VSMCs, causing vascular relaxation in adult vasculature [35]. The endothelial isoform of nitric oxide synthase (eNOS) diffuses from EC to VSMCs, where it activates specific protein kinases, and initiate VSMCs relaxation [36]. However, when endothelial dysfunction occurs, it is associated with a decrease of NO bioavailability and variations in the release of vasoactive compounds [37,38]. NO derived from EC has also been reported to change flow-dependent vascular remodeling associated with a negative regulation of Platelet derived growth factor (PDGF) [39]. NO from EC appears to regulate the metabolic activity of the enzyme aldose reductase in VSMCs and preventing sorbitol accumulation of diabetic rat aorta [40]. Additionally, Carbon monoxide (CO) is indirectly connected with the NO vasodilative role in EC–VSMCs crosstalk. Transient HO-1/CO-regulates vascular tone via upregulation of the eNOS/NO axis in ECs [41] and subsequently increases cGMP production in VSMCs [42]. EC enhancing phosphorylation of eNOS, and production of NO, has been also involved in autophagy. Adaptative autophagy within the endothelium and VSMC has been described as an important mechanism in maintaining vascular function in vascular remodeling diseases [43], and in cardiac microvasculature [44,45].

Crosstalk studies have described that ECs also release other factors such as Angiotensin II or endothelin involved in the VSMCs contraction and increasing of the vascular tone [35,46]. ECs also respond to mechanical force stimulus to control the growth of the underlying VSMCs. Changes in the hemodynamic forces contribute to the regulation of endothelial production of soluble heparan sulfate proteoglycans (HSPGs). These molecules are secreted by ECs and inhibit VSMCs proliferation upon mechanical strain [47]. In this sense, perlecan expression is regulated by mechanotrasduction in EC and is essential in the control of VSMCs proliferation by altering their response to transforming growth factor β (TGF-β) in an ex vivo system for culturing aortic rings [48]. Perlecan also inhibits VSMCs proliferation in vivo in a mice model of intima hyperplasia [49,50].

Platelet-derived growth factor (PDGF) is other of the numerous growth factors that is essentially implicated in the EC–VSMCs communication, either in physiological or pathological situation. This molecule is produced by VSMCs, activated macrophages, and EC [51]. During embryogenesis, endothelial-PDGF is involved in the VSMCs recruitment, proliferation and migration, all of them processes necessaries for the correct assembly and formation of the vessel wall [52]. However, different circumstances could alter the response of VSMCs to the EC-secreted PDGF-B in mature vasculature. For example, studies in ECs and VSMCs cocultures have demonstrated that shear stress is converted into an intracellular signal of PDGF-BB up-regulation in the dysfunctional endothelium and triggers migration and proliferation of the underlying VSMCs [53]. While the role of PDGF-BB is related to the paracrine control of VSMCs phenotype by ECs, other growth factors such as Tissue growth factor B1 (TGF-β1) have been described participating in the feedback control from VSMCs to ECs. [54]. Shear stress signal is mechano-transduced in upregulation of TGF-β1 expression in ECs. TGF-β1 expression by ECs in a 3D EC–VSMCs coculture model demonstrated that could modulate features of pathological vascular remodeling, such as, VSMCs phenotypic switching and VSMCs extracellular matrix synthesis [55]. Moreover, some evidences have described a new cellular crosstalk concept in which synthetic VSMCs phenotype induced by PDGF-BB displayed an anti-angiogenic effect in ECs, whereas contractile VSMCs phenotype promoted a pro-angiogenic activity in ECs [56]. Moreover, aberrant secretion of PDFG by ECs induces VSMCs proliferation and migration in injured artery by hyperhomocysteinemia (HHcy). In this regard, human EC–VSMCs cocultured studies have demonstrated that high level of HHcy promotes proliferation and migration of VSMCs due to a DNA demethylation of PDGF in ECs. Upregulation of PDGF was confirmed in the aortic intima of mice with HHcy [57]. Moreover, in vivo model of neointimal hyperplasia has recently demonstrated that endothelial cell-specific regulation of PDGF-B modified VSMC phenotypic state and neointima formation [58].

PDGF-B axis has been defined as a paracrine endothelium-to-mural vascular cells signaling loop. In contrast, one of the main representative pathways in the opposite orientation—from VSMC to the endothelium—is the angiopoietin-Tie receptor axis. The angiopoietin (Ang) family of secreted growth factors interacts with Tie receptors, which are expressed throughout the developing embryonic endothelium and in the quiescent adult vasculature [59]. Between them, tyrosine kinase Tie-2 receptor and angiopoietin 1 (Ang-1) secreted molecule, act as endothelial cell survival factors, promoting stabilization of blood vessels and regulating vascular remodeling through EC–VSMCs communication [60]. During vascular remodeling, it has been described as an increase of Angiopoietin-2 (Ang-2) secretion in tissue, which inhibits the Ang1-induced Tie2 activation through competition for the same receptor. This antagonistic Ang-1/Ang-2 concept in which Ang-2 was identified as the main destabilizing of the quiescent endothelium by an internal autocrine loop mechanism was corroborated in vitro in a 3D EC–VSMCs coculture model [61]. In the same manner, the Ang-2 role is associated with disease pathologies such as microaneurysms and hemorrhages of the retinal blood vessels in diabetes [62], and early stages of fatty streak in atherosclerosis [63]. However, in vivo studies on mice model of AAA and atherosclerosis provided evidences of the protective role of Ang-2, suggesting the necessity of deeper studies of this axis in the EC–VSMCs crosstalk [64].

The sphingosine-1-phosphate (S1P) pathway is another receptor-ligand axis involved in the paracrine communication between EC and VSMCs [65,66]. S1P signaling in EC has secondary consequences for EC–VSMCs interactions. SP1 is a sphingolipid metabolite that signals through a family of G-protein-coupled receptors [S1P(1–5)].It has been shown that deletion of the S1P1 receptor on ECs results in significant defects in VSMCs coverage, in a human coculture model of EC and VSMCs. These data have suggested that endothelial SP1 promotes the expression of the inhibitor of metalloproteinase TIMP-2 in VSMCs, contributing to the incomplete formation of endothelial cell adherent’s junctions [67].

Furthermore, the data found for the vascular mammalian target of rapamycin (mTOR) pathway represent another level of EC–VSMCs crosstalk, which support the necessity of coculture experiment to have a global vision of the pathology. In this sense, in a model of EC/VSMCs vessel-like construction, the interaction of VSMCs with ECs regulated the response of the EC to flow and injury. This mechanism is described due the regulation of mTOR expression in ECs. This in vitro work demonstrated that presence of VSMCs post-stent could be necessary for regulates endothelial recovery, and may explain the possible negative impact of local targeted VSMCs antiproliferative stent-delivery [68].

In summary, despite the fact that paracrine secretion occurs during the endothelial dysfunction in pathological situation, few studies have addressed how are the changes in the cell–cell conversation directly produces by this endothelial dysfunction.

**- Extracellular vesicles**

Secretion of extracellular vesicles (EVs) is an important mechanism by which the intercellular communication is taking place, either in vascular homeostasis or during pathological vascular remodeling [69,70]. EVs serve as intercellular messengers and they could be classified in exosomes, microvesicles and apoptotic bodies, depending of their size, lipid composition, or mechanisms of formation and discharge [71]. The smallest EVs are the exosomes, by contrast, apoptotic bodies share the biggest size with more than 1 µm. Apoptotic bodies and microvesicles are formed by cytoskeleton rearrangement and are exerted from cells through direct plasma membrane blebbing. Exosomes are generated by endosomal pathway leading to the inward budding of multivesicular bodies (MVBs). Normally, under different physical and/or pathological stimuli, the plasma membrane inward buds and produces endosomes (EEs) [72]. In the latest states, endosomes induce the formation of the multivesicular bodies (MVBs) by the machinery of the endosomal complex, which will accumulate intraluminal vesicles (ILVs) in their inner spaces. MVBs could suffer different dynamic alterations depending on the stimulus and, while ones MVBs could be degraded by the proteasome, others could be secreted by fusion with the plasma membrane as “exosomes”. These exosomes could be loading by different functional cytosolic components such as microRNAs (miRNA), and mRNA that could be released to extracellular space and regulate cell communication constituting a novel means of cell–cell communication [73]. miRNAs are small single-stranded noncoding RNAs (average ≈18–24 nucleotides) evolutionarily conserved that have been emerged as regulators of pathophysiological cellular processes [74]. In this regard, mechanisms involved in the vascular remodeling diseases such as, proliferation, lipid uptake and efflux, cellular adhesion, or inflammation, are only an example of the important mechanisms that could be controlled by miRNAs [75]. miRNAs regulate gene expression at the post- transcriptional level, binding to the 3′-untranslated region (UTR) of a specific target mRNA sequences, triggering the reduction of protein expression by impeding the translation and promoting mRNA degradation [76]. Moreover, many studies have described miRNAs as potential biomarkers for diagnosis, prognosis, or therapeutics targets in CVD, since they are circulating in blood and in other body fluids (plasma, saliva, urine, bile, and breast milk) contained within exosomes [77]. In relation with EC–VSMCs interaction, recent studies have demonstrated that EC and VSMCs release extracellular vesicles that could contribute to vascular homeostasis or pathological progression. In physiological situation, the normal laminal-flow triggers the EC release of miR-143 and miR-145 containing in extracellular microvesicles. miR143/145 are the highest expressed miRNAs in normal vessel wall and have been involved in the cell-to-cell communication, necessary to maintain the contractile functional capacities of the vascular cells in vessel wall [78]. In this sense, the atheroprotective role of these miRNAs has been demonstrated in vitro in cocultures experiments. ECs packaged and released miR143/145 into exosomes, that are taken-up by neighboring VSMCs, contributing to regulate phenotypic target genes in these cells [79]. Furthermore, miR-206 expression in human umbilical endothelial cells (HUVEC) is involved in the regulation of the contractile phenotype of VSMCs (α-SMA, Smoothelin and Calponin expression genes) by suppressing exosome secretion from endothelium [80]. However, in response to vascular injury, the miRNAs that are contained in the EVs could be modified. The expression of the miR-143/miR-145 cluster is markedly reduced in injured arteries compared with normal vessel, favoring the phenotypic switching of VSMCs. This reduction triggers transcriptional regulation of Krüppel-like factor (KLF)-4-5 dependent’s genes, essential for contractile VSMCs phenotypic switching to synthetic and proliferative phenotype. miR-143/ miR-145 have been also shown to target other key transcriptional regulators implicated in modulation of VSMCs differentiation such as ETS domain-containing protein-1 (ELK-1) and myocardin [81,82]. Intravenous delivery of miR-143/145 extracellular vesicles blocked atherosclerotic lesion progression [83] and analogous protective effects have been observed in response to neointimal lesion formation [84]. However, it remains to be determined whether bidirectional extracellular miRNA passage occurs between VSMCs and EC under atherosclerotic conditions. A recent work has demonstrated that EC regulates VSMCs phenotype via inflammasome-dependent EVs [85]. Moreover, a different study showed that physiological laminar flow also increases the release of endothelial-miR-126. EC-miR-126 serves as a mediator for the maintenance of VSMCs contractile phenotype by the regulation of VSMCs target gene such as FOXO3, B-cell lymphoma 2 (BCL2), or insulin receptor substrate 1 [86]. The function of miR-126 in the physiological EC–VSMCs communication was also supported by its atheroprotective effects in vivo [87]. However, it has been showed that changes in the levels of miR-126 from ECs could serve as messenger to VSMCs to proliferate under pathological shear stress [86]. In contrast to the functions of endothelial miR-143/miR-145 or miR-126 that are expressed in physiological condition to maintain VSMCs contractile phenotype, many of miRNAs are secreted from cells under pathological condition. For instance, such is the case of miR-221 and miR-222 that are increased in VSMCs in response to neointimal injury [88], where they may play a destabilizing role in atherosclerotic lesions [89]. Studies of exosome-mediated crosstalk under pathological condition have recently demonstrated that, VSMCs secreted miRNA-loading EVs works as messenger to ECs in pathological circumstances. In this sense, TGF-β1 decreases exosome production while PDGF-BB increased it. Moreover, oxLDL upregulates exosome-mediated transfer of miR-155 in human VSMCs (HASMCs) in a mechanism dependent of Krüppel-like factor 5 (KLF5) [90]. miR-155 upregulation from VSMC is received by the ECs and it inhibits endothelial proliferation/migration and re-endothelialization, either in vitro or *in vivo,* increasing vascular endothelial permeability [90]. Similar results on endothelial migratory activity were described in artery endothelial cells (PAECs) exposed to PDGF stimulated VSMCs-derived exosomes. Downregulation of miR-1246, miR-182, and miR-486 in vascular smooth muscle´s exosomes promote endothelial migration [91]. Moreover, VSMCs secrete exosomes that promote vascular calcification under calcium stress triggers [92,93].

### 2.2. Parenchyma Players (Interaction via the Extracellular Matrix(ECM))

Cell communication could also be through the extracellular matrix (ECM) properties. Alterations in ECM not only cause structural modifications, but also could lead to EC–VSMCs signaling disruption [94].

Both ECs and VSMCs secrete matrix components that contribute to the maintenance of vessel properties and influence neighboring cell functions. Furthermore, ECM serves as a source and reservoir of signaling mediators, that is changed depending on the period of vessel wall development. The reservoir potential of ECM has been described as a way of cell communication in the regulation of cell growth, plasticity or metabolism [95,96]. Some of the best characterized ECM binding proteins, whose contribute to cell signaling are; latent (TGF-B binding protein (LTBP 1–4), emilins, microfibril associated glycoproteins (MAGP-1 and 2), and members of fibulin family [97]. The relationship between ECM and the resident cell is reciprocal, and changes in physical forces are detected by matrix-binding cell receptors and mechano-transduced in cell signals [96]. Among the ECM components, collagen is the main molecule that contributes to the geometric changes in the vessel wall, and whose deposition favors the vessel stiffness [97,98], and may serve to connect basement membrane of VSMCs and EC with other ECM structures. Collagen has pleiotropic effects on VSMC phenotype depending on the type of collagen deposited. Furthermore, while collagen I and fibronectin induce synthetic VSMCs phenotype [99,100], secretion of collagen type-IV by cells promote a contractile VSMCs phenotype signal transduction [99,100,101,102,103,104,105].

### 2.3. Contact-Contact Signaling

Ultrastructural studies have described close contact sites by which EC and VSMCs communicate in blood vessels. Such direct contact sites facilitate metabolic and electrical coupling conduits, and transport of signaling molecules between EC and VSMCs. Biologically, these contact sites permit direct bidirectional communication of molecules and ions between adjacent cells as a key pathway for coordinating vascular function [106]. There are different types of direct structural connections;

**- Myoendothelial gap junctions (MEGJ)**

The most studied cell–cell contact is called Myoendothelial gap junctions (MEGJ) [107]. These are direct contacts through the fenestrated internal elastic lamina (IEL), which are the result of the actin-based cell protrusions (either from EC, VSMCs or both) [108]. The formation of the functional gap junctions requires the assembly of the connexins (Cx) proteins. In the vascular system, Cx37, Cx40, Cx43, and Cx45 are the major connexins described [109]. The presence of these projections seems to vary depending on the vessel type and conditions. So, while in small mesenteric arteries, MEGJ are located at the interface of EC projections and the surface of VSMCs, in large arteries MEGJ are also at the interface of adjacent cells (either between homotypic EC-EC or VSMCs-VSMCs) [107]. Furthermore, the composition and quantity of MEGJ are condition-dependent, and could be differentially regulated in EC or VSMCs by posttranslational modifications [110]. Essentially, MEGJ serve as feedback pathways between VSMCs and EC to facilitate the direct transfer of ion or small molecules, mainly second messengers such as Ca^2+^, IP3, and cAMP [111,112], or endothelium-derived hyperpolarization (EDH) signals, to control the vascular constriction-relaxation [113]. Vascular remodeling pathologies such as atherosclerosis have been associated with changes of endothelial Cx expression and function. In this sense, ECs from atherosclerotic plaques do not express Cx37 and Cx40, while endothelial Cx43 expression is induced [114]. Moreover, the atherogenic stimuli oxLDL increases Cx43 phosphorylation, which is associated with reduced coupling between EC and VSMCs [115]. Furthermore, oxidized phospholipids regulate Cx43 expression in VSMC, associated to phenotypic changes, in a model of atherogenesis in ApoE deficient mice [116]. Additionally, low shear stress occurring during atherosclerosis also altered endothelial Cx40/Cx43 expression [117]. Interestingly, in vitro experiments have shown that the main inflammatory mediators found in vascular remodeling, such as lipo-polysaccharides (LPS), tumor necrosis factor-α (TNF-α), and interleukin-1β (IL-1β), also inhibit human MEGJ affecting EC–VSMCs communication [118].

**- Notch signaling**

Strong evidences have demonstrated the role of Notch signaling in the EC–VSMCs crosstalk [119]. Notch is an evolutionary-conserved cell-to-cell signaling mechanism crucial in vasculogenesis, due to it is involved in artery, vein, and capillary organization and development. Notch signaling activation needs the interaction of the membrane-bound Notch receptors (Notch 1–4), to the membrane-bound Notch ligand (Jagged1, 2 and Delta-like 1, 3, and 4) of the adjacent cells [120]. Both, EC and VSMCs ubiquitously express Notch elements. However, Notch elements suffer changes dependent of the cell stages and cell type. The main function of Notch in the EC–VSMCs communication is, partly, driven by Jagged1 ligand. The endothelial expression of Jagged1 is necessary for the proper and complete arterial VSMCs maturation. In this regard, in vitro coculture studies have demonstrated that, ECs-expressed Jagged 1 induce Notch3 expression in VSMCs, which is critical for cell differentiation and acquisition of mature arterial identity [121,122]. In addition, VSMCs and ECs coculture experiments have shown that EC growth in response to angiogenic stimuli is modulated by VSMCs through Notch signals [123].

Modulation of Notch signaling has not been only associated with physiological EC–VSMCs communication, but also to pathological vascular remodeling disease. Moreover, impair of Notch3 expression is phenotypically related to enlarged arteries, with aberrant distribution of elastic laminae, and VSMCs with venous appearance [124]. These defects in Notch3 receptor lead to a non-functional vessel wall, formed by non-fully contractile VSMCs [125], and is responsible of some disorders such as familiar aortic valve disease or cerebral autosomal dominant arteriopathy with subcortical infarcts and leukoencephalopathy [126]. Alteration in endothelial Notch activation was also associated with a protective role in atherosclerosis [127], and an important role in controlling VSMCs phenotypic switching in vascular injury [128]. Moreover, recent works have also shown evidence on the role of EC–VSMCs crosstalk by Notch signaling in the formation of the atheroma fibrous cap.

Another example of EC–VSMC contact interplay is the interaction between the membrane-bound Eph receptor tyrosine kinase (Eph) with the membrane-bound Ephrin ligands. The well-studied receptor-ligand pair EphB4 and ephrin-B2, are reciprocal expressed on EC or VSMCs and are required during embryonic vessel development, vascular remodeling, and pathological vessel formation in adults [129,130]. Moreover, Ephrin-B2 regulates EC–VSMCs crosstalk by VEGF receptor endocytosis in VSMCs [131]. Although their role in EC–VSMC crosstalk needs to be established, Ephrin-B2 is expressed in atherosclerotic plaques colocalizing with EC, which could suggest a potential role in the disease [132].

## 3. In Vitro Models to Study EC–VSMCs Communication during Vascular Remodeling

The importance of understanding the EC–VSMCs communications in vascular remodeling diseases have led to great efforts in developing relevant in vitro models. It has been essential to create a quiescent EC–VSMC coculture modeling in which could be possible to recapitulate the flow dynamic, the environment, or the structure, trigging to mimic the in vivo vessel wall circumstances [133]. The approaches to study EC–VSMCs communications have been done in a variety of ways; starting with independent cultured cells treated with conditional cell medium from other cell, carrying on with 2D coculture, which provides a simple way of studying both cell types together, and ending with the use of 3D coculture, vasculature on-chip models, and organoids. Although, some of these models appeared to mimic the distinct aspects of the vascular remodeling, the complexity and the multimodal nature of the EC–VSMCs communication that has been dissected before made difficult to generate a common in vitro coculture model. Moreover, the morphology and polarization of cells within the cocultures, together with the formation of an extracellular matrix, and the quiescence phenotype are other limiting factors. The necessity to generate a common and well-stabilized in vitro model of EC–VSMCs coculture is considered necessary and critical for future studies and for translational drug screening. Types of cocultures could be classified in:

**- Indirect coculture:**

In vitro model to investigate cell–cell dependent interaction without physical contact, and based in secretory pathways and in paracrine communications. Microcarrier, scaffold, bilayer membrane, use of conditioned media (CM) or transwell assay are examples of indirect coculture.

Conditioned media; In this model, cells are grown separately and the medium of one of them is used to stimulate the other cell type. However, the unidirectional response, and the soluble factors being the only mediators studied without cell–cell interaction, are some of disadvantages of the model. This model has been extendedly used in different studies related with atherosclerosis. However, more bibliography around the model of CM using VSMCs and immune cells has been achieved, than to study the potential relationship between EC–VSMCs. In term of VSMCs-immune cells interaction, features of progression of atherosclerotic plaque has been described, such as, the role of macrophages-soluble mediators on; VSMCs calcification [134,135], formation of lipid laden VSMCs [136], vascular proliferative and migrative capacities [137,138,139] or ECM composition and neo-angiogenesis [140].

Two-dimensional (2D) cell cultures EC–VSMCs (transwells, flow chambers). This methodology has been widely used, and it has the advantages of being technically simple, whilst enabling microscopy examination, and easy isolation of pure populations without cell sorting. Briefly, the methodology consists in one cell type cultured on the bottom of the plate and the other one on the membrane filter. Distance and filter prevent cell–cell contact, although some variants of the model admit cell culture at different sides of the filter membrane [141]. Moreover, the variability of the membrane pore size permitted in some case the interaction between ECs and VSMCs and allows this in vitro model to be rutinary used in the vascular remodeling studies to assess, migration, immune cell transendothelial, permeability studies, or cell–cell paracrine regulation [28].

**- Direct Coculture**

The main limitation of indirect cocultures is the lack of cell–cell contact that is experimented by cells in the vessel wall. It is not the case of the 3D models. Different variants of 3D coculture models could allow one to study the three main types of cell interaction that have been described previously: cell–cell contact, paracrine interaction, and cell-ECM interaction, making up these models physiologically more relevant to study the pathological and physiological vascular remodeling. Some examples of 3D coculture systems are: EC–VSMCs spheroids, including direct cultures of ECs and VSMCs, or cultures of ECs on extracellular matrix-like gels containing VSMCs (collagen scaffolds), and vascular organoids created from induced pluripotent stems cells (IPs).

The spheroids model is based on the generation of suspended cell spheres using differentiated cells. Studies in EC spheroids have described that oxidized phospholipid [142] or supernatants of activated natural killer (NK) cells [143] promoted growth of capillaries in the context of atherosclerosis lesion progression. Recently, the assessed of a spheroid model of human endothelial and vascular cells has been used to study the impact of ECs on the gene expression pattern of VSMCs [105]. In this model, different source of human EC and VSMCs were cultured in suspended cell spheres, which spontaneously organized into a central composite VSMCs core enclosed by a human EC monolayer. Interestingly, 3D interaction of ECs with VSMCs in this model downregulates expression of smooth muscle-genes involved in cholesterol biosynthesis [144]. Moreover, the same spheroids model, instead using myeloid cells from blood and myofibroblasts, was established to study late atherosclerotic lesion (fibroatheroma) in vitro. Authors described how LDL affect cell viability and contribute to population polarization in fibroatheroma [145]. Although, this kind of model cannot address the complexity of the human vascular environment (shear stress, LDL–cholesterol variation, blood pressure etc.) it provides a tool for investigating cellular interplay, sharing anatomopathological features with human native plaques. In addition, other studies have tried to integrate relevant physiological factors into a vascular disease-mimicking tissue, resulting in a 3D tubular artery-like constructs formed by collagen-rich extracellular matrix (as the tunica externa), VSMCs (as the tunica media), and an EC lining (as the tunica interna). This new approach open questions in relation to the gel scaffold used, the heterotypic cell–cell contact, or discrepancies in cell metabolic trends in planar and tubular growth environments [146].

Although, these pre-clinical studies in vitro could eventually be used, unfortunately, in many occasions failed to translate it into clinical efficacy, indicating that novel pre-clinical systems are needed. Next step of complexity in the in vitro modeling is the 3D human-derived blood vessel organoids described by Wimmer R 2 years ago [147]. This model represents a promising “humanized bridge” for a proper pre-clinical research. Human vascular organoids consist in the induction of pluripotent stem cells (hPSC) aggregates, and subsequent differentiation into endothelial and pericytes in a 3D collagen I–Matrigel matrix, to establish vascular networks [147]. These in vitro 3D human blood vessel organoids are formed by self-organization, and exhibit morphological, functional, and molecular features of human microvasculature. This system has been used to recapitulate the structure and function of human blood vessels to study the regulators of vasculopathy diseases [148], as in the case of diabetic vasculopathy [149]. Moreover, similar approaches of human blood vessel organoid have been used to understand the way of entrance of SARS-CoV-2 infection in vascular cells [150]. In the future, the application of organoids will open the door to new approaches of drug screening. Interestedly, the opportunity to establish new human models of aortic diseases with the use of patient-iPSCs will offer possibilities for a personalized medicine in some clinical fields.

Although, 3D models represent vessel wall architecture, these models do not recapitulate the mechanical conditions of the native tissue, related with the hemodynamic forces. Moreover, these hemodynamic conditions are necessary to determinate the cellular alignment and organization of the native arterial vessel wall. Changes in blood flow are critical in vascular remodeling and in EC–VSMCs organization and interaction. The ability to integrate multiple cell types and flow systems in microfabricated devices enables tissue engineering to introduce various atherogenic features. In this sense, a few numbers of works have tried to generate dynamic 3D models under hemodynamic environment. Thirty years ago the first in vitro gel-based coculture model for atherosclerosis allowed to study the mechanism behind LDL-mediated macrophages transmigration using a gel-separation coculture of EC and VSMCs [151]. Interestingly, other modalities of this model exist to assess the interaction of mural cells (EC–VSMCs) with immune cells, and are also subject to shear stress [130]. This last model revealed that hemodynamic shear stresses served as modulator of the EC phenotype, and also played a critical role in transcriptional regulation of the VSMCs phenotype [152,153]. A model system with sequentially layered VSMC/EC vessel-like constructs connected to a perfusion bioreactor have been used to recapitulate physiological flow in relevant studies of vascular intervention, including bolus drug administration, balloon deployment, and stent implantation [154]. Other model of three-dimension coculture system in a stretchable microfluidic device was used to address the effects of stretch and LDL on foam cell formation. It is based on three layers of polydimethylsiloxane membrane capable of delivering nonuniform strain over EC, VSMCs, and immune cells cultured on it [155]. This model of the hemodynamic EC–VSMC signaling niche provides a controlled micro-platform to study EC–VSMC signaling in a physiological or pathophysiological perturb hemodynamic environment, reflecting more appropriately the tissue organization in vivo. Finally, EC–VSMC-signaling-on-a-chip allows co-culturing of human aortic EC and aortic VSMCs, separated by a porous membrane, which enables EC–VSMCs interaction and signaling under hemodynamic conditions [156]. Moreover, other groups have tried to mimic hemodynamic and ECM stiffness factors during atherosclerosis. Collagen-based hydrogel matrices with different densities have been employed to grow cells and recapitulate the porosity of early (low tissue density) and advanced (high tissue density) atherosclerotic plaques [157]. Moreover, another microfluidic model was described to study the earlies stages of atherosclerosis and foam cell formation, in which EC and VSMCs cocultured in elastic membrane and embedded in the microfluidic device are exposed to low-density lipoprotein and stretch [155].

## 4. Conclusions, Clinical Implications, and Future Directions

**Figure 1:**
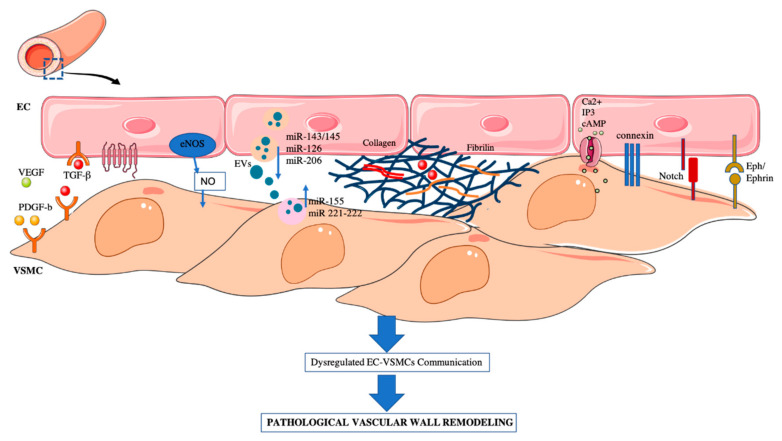
EC–VSMCs communication in a vessel wall. Schematic representation of: paracrine, extracellular vesicles, ECM and direct cell–cell interaction. Dysregulation of EC–VSMC communication triggers the pathological vascular wall remodeling associated to some CVDs.

Communication between mural cells in blood vessels is fundamental for the correct formation and function of the vasculature. In this review we have summarized how the physical or paracrine EC–VSMCs communication give rise to the vascular-bed-specific characteristics (Table 1), and how changes in this crosstalk are the main cause of pathological vascular remodeling (Figure 1).

Pathological vascular remodeling situation is usually asymptomatic and gives the face by a late clinical event. Human samples coming from tissues biopsies, provides limited information about the development of the lesion, and show only the tip of the iceberg of the vascular injury. For that reason, further research should be directed at strengthening the current understanding of interaction between VSMCs and ECs in co-culture models. This could provide a major source of knowledge about vascular homeostasis in order to find novel therapies in the prevention of vascular remodeling progression. Proteomic studies in human tissue, conditional medium, or serum of patients with vascular remodeling (atherosclerosis, AAA etc.) have revealed a huge battery of potential diagnostic markers and are good approaches to screening for therapeutic targets [158,159,160,161]. Furthermore, in human plasma, human EVs plasma levels are increased in individuals with higher risk of cardiovascular events such as hypertension [162]. In addition, proteomic studies of the exosomes cargo of patient serum have revealed promising biomarkers for remodeling pathologies. Some studies have illustrated how the presence of miRNAs or other molecules in circulating EVs of patient could acts as a type of messenger or signaling molecule, and could serve as biomarkers of CVD [163]. Moreover, EVs, and especially exosomes, possess some properties which are good for therapeutic delivery such as biocompatibility, biological barrier permeability, low toxicity, and low immunogenicity [164]. However, the development of in vitro vessel wall modeling would reduce costs, variability, and eventually allow for high-throughput molecular and drug screening. In addition, development of organoid systems will improve our understanding of molecular mechanism and could serve as a personalized therapy tool for vascular remodeling diseases patients.

## Figures and Tables

**Table 1:** Summary of the EC–VSMC crosstalk, define by different in vitro models.

EC–VSMC Communication		Methodology	Molecule/Signal Pathway	Results	References
Paracrine	Soluble Factors	EC–VSMC coculture in opposite sides of transwell	↑VEGF, PDGF-AA, PDGF-BB, and TGF-β in VSMCs ↓bFGF	Coculture affect gene and protein expression of angiogenic factors	[26,27,28]
		Conditioned culture media	↑TF	EC suppress the proliferation of co-existing VSMCs	[29]
		Coculture flow chamber system	↑ICAM-1, VCAM-1 and E-selectin gene expression	Under static conditions, coculture with VSMCs induces adhesion proteins expression in ECs	[30]
		Coculture flow chamber system	↑GRO-α, MCP-1	Under static conditions, coculture with VSMCs induces GRO-α, MCP-1 in ECs	[31]
		Microcarrier coculture system	LDL	EC influenced VSMC’s LDL metabolism	[33,34]
		Conditioned culture media/Ex vivo aortic ring	eNOS, cGMP, endothelin, AngII	Regulation of the vascular tone	[33,39,40,46]
		Ex vivo aortic ring	Perlecan	Mechanotransduction in EC controls VSMCs proliferation	[48]
		Coculture flow chamber system	PDGF-BB	EC triggers proliferation and migration of VSMCs Synthetic VSMCs modulate anti-angiogenic effect over EC	[53,54,56]
		Coculture flow chamber system	TGF-β1	EC modulates VSMCs phenotypic switching and extracellular matrix synthesis	[55]
		Spheroids coculture	Ang-1/Ang-2	Desestabilization of the quiescent endothelium	[61]
		In vitro model of a vessel-like construct	mTOR	VSMCs regulates EC response to flow and injury	[68]
	Extracellular vesicles	Conditioned culture media/Boyden chamber assay	miR143/145 miR-206 miR-126	Endothelial EVs regulate VSMCs phenotypic changes	[79,80,81,82,83,84,85,86]
		Conditioned culture media/Boyden chamber assay	miR-221/miR-222 miR-155 miR-1246, miR-182, miR-486	VSMCs EVs regulate endothelial permeability, migration and vascular calcification	[90,91,92,93]
Parenchymal players		3D Bioprinted gelatin hydrogel platform	Collagen I, IV, fibronectin, heparan sulfate chains	Extracellular matrix presentation modulates VSMCs mechanostransduction	[103,104,105]
Direct contact	Myoendothelial gap junctions (connexins)	EC–VSMCs coculture in opposite sides of small pore transwell	Second messengers (Ca^2+^, IP3, camp)	Vascular constriction-relaxation. Phenotypic changes	[113,114,115,116,117,118]
	Notch signaling	EC–VSMC coculture in opposite sides of small pore transwell Human-derived blood vessels organoids	Notch3 receptor BMPR2-Notch1 DII4 and Notch3	VSMCs phenotypic switching, EC regeneration and maintainer of EC monolayer integrity Regulators of diabetic vasculopathy	[125,126,127,128,147,148,149]
		Spheroids coculture	Ephrin-B2	VSCMs migration and EC adhesion	[129,130,131]
		Spheroids coculture	24-dehydrocholesterol reductase	EC control VSMCs cholesterol levels	[144]
		3D tubular artery-like constructs	Glucose metabolism	Investigation of late atherosclerosis lesion	[145,146]

## Data Availability

Not applicable.
